# Redrawing Urokinase Receptor (uPAR) Signaling with Cancer Driver Genes for Exploring Possible Anti-Cancer Targets and Drugs

**DOI:** 10.3390/ph16101435

**Published:** 2023-10-09

**Authors:** Yu-Ching Chang, Chung-Ze Wu, Chao-Wen Cheng, Jin-Shuen Chen, Li-Chien Chang

**Affiliations:** 1 Graduate Institute of Life Sciences, National Defense Medical Center, Taipei City 114201, Taiwan; yc.chang112917@gmail.com; 2 Division of Endocrinology and Metabolism, Department of Internal Medicine, School of Medicine, College of Medicine, Taipei Medical University, Taipei City 110301, Taiwan; chungze@tmu.edu.tw; 3 Division of Endocrinology and Metabolism, Department of Internal Medicine, Shuang Ho Hospital, Taipei Medical University, New Taipei City 235041, Taiwan; 4 Graduate Institute of Clinical Medicine, College of Medicine, Taipei Medical University, Taipei City 110301, Taiwan; ccheng@tmu.edu.tw; 5 Graduate Institute of Medical Sciences, National Defense Medical Center, Taipei City 114201, Taiwan; 6 Department of Education and Research, Kaohsiung Veteran General Hospital, Kaohsiung City 813414, Taiwan; 7 Division of Nephrology, Department of Internal Medicine, Tri-Service General Hospital, National Defense Medical Center, Taipei City 114202, Taiwan; 8 School of Pharmacy, National Defense Medical Center, Taipei City 114201, Taiwan

**Keywords:** uPAR-mediated signaling system, cancer driver gene, network analysis, uPAR modulator, a pharmaceutical bioinformatics study

## Abstract

During tumorigenesis, urokinase (uPA) and uPA receptor (uPAR) play essential roles in mediating pathological progression in many cancers. To understand the crosstalk between the uPA/uPAR signaling and cancer, as well as to decipher their cellular pathways, we proposed to use cancer driver genes to map out the uPAR signaling. In the study, an integrated pharmaceutical bioinformatics approach that combined modulator identification, driver gene ontology networking, protein targets prediction and networking, pathway analysis and uPAR modulator screening platform construction was employed to uncover druggable targets in uPAR signaling for developing a novel anti-cancer modality. Through these works, we found that uPAR signaling interacted with 10 of 21 KEGG cancer pathways, indicating the important role of uPAR in mediating intracellular cancerous signaling. Furthermore, we verified that receptor tyrosine kinases (RTKs) and ribosomal S6 kinases (RSKs) could serve as signal hubs to relay uPAR-mediated cellular functions on cancer hallmarks such as angiogenesis, proliferation, migration and metastasis. Moreover, we established an in silico virtual screening platform and a uPAR–driver gene pair rule for identifying potential uPAR modulators to combat cancer. Altogether, our results not only elucidated the complex networking between uPAR modulation and cancer but also provided a paved way for developing new chemical entities and/or re-positioning clinically used drugs against cancer.

## 1. Introduction

The urokinase-type plasminogen activator (uPA) system embodies ligand uPA, receptor uPAR and inhibitors PAI-1 and PAI-2. It goes through its sole mediator, uPAR, a glycosyl phosphatidylinositol (GPI)-anchored membrane protein hinging three homologous cysteine-rich domains (DI, DII, DIII), to modulate many physiological/pathological processes [1]. The signaling of this system uses two different methods to transduce. In a proteolytical fashion, the untie of uPAR’s extracellular ligand-binding portion of DI and DII-DIII fragments into circulation by proteases (such as uPA) seems to serve as an autocrine signal in extracellular proteolysis and is delineated to tumor progression and inflammation [2,3]. In a non-proteolytical fashion, the uPA-bound uPAR complexing integrins to interact with receptor systems, such as receptor tyrosine kinases (RTKs) (e.g., EGFR, FGFR, PDGFR, IGFR), G protein-coupled receptors and formyl peptide receptor type 1 (FPR1) [4], is thought to behave as an intracellular signal involved in angiogenesis, cell movement and tumor progression [5,6]. In addition, uPAR is rarely expressed in normal tissues but overexpressed in some tumor tissues; these traits grant its role in diagnosis and prognosis of various malignancies [2,7]. Together, the tangling between the uPAR signaling and cancer is tight and intricate.

The foregoing relationships serve to elucidate the detail that the uPAR signaling may have a great impact on cancer biology and therapy. Some previous works targeting uPAR by monoclonal antibody, peptide-derived antagonists and small molecule inhibitors (reviewed in [8,9]), which antagonize the binding with uPA or vitronectin (Vn), showed promise at preclinical stages but failed to receive clinical approvals. On the other hand, targeting modulation of the uPAR non-proteolytic signaling to combat cancer has not been truly established due to the complexity of this intracellular signaling because the uPAR signaling remains unresolved in the post-genomic era. It is well known that several types of cancer hallmarks, such as proliferation, angiogenesis, invasion and metastasis, are affected by the uPAR signaling. Thus, how this system governs cancer cells to acquire aberrant phenotypic capabilities during malignant processes must be elucidated in order to break the deadlock in the development of uPAR-related anti-cancer agents.

Cancer driver genes are genes whose mutations can fuel cancer progression and lead to pathological changes in tumor microenvironment, angiogenesis and inflammation. Although alternative definitions and search methods are used to identify and catalogue cancer driver genes and mutations, all these genes are mutated in at least one cancer type. Therefore, cancer driver genes may represent the spot genes in any physiological signaling system that drive the oncogenicity. On the other hand, while continuous efforts based on sophisticated techniques have broadened our knowledge on this topic, many studies indicate that driver genes are often “hub” genes, playing a key role in managing complicate signal transduction. Since cancer driver genes may play the pivotal role in uPAR signaling, we propose to map out this signaling pathway with cancer driver genes for not only clarifying the crosstalk between uPAR signaling and cancer but also providing the intersection drivers in uPAR signaling as druggable hubs for developing small molecule modulators to treat cancer.

In this study, a pharmaceutical bioinformatics framework aimed to elucidate cancer driver-associated uPAR signaling via network construction, pathway analysis, machine learning and model validation was proposed. With the use of self-defined uPAR modulators to construct the uPAR signaling network via linking uPAR co-expressed cancer driver genes, the rationale for uPAR modulators as a possible strategy to reverse cancer hallmarks was verified. Understanding the cancerous uPAR signaling networks could lead to the development of a new class of anti-cancer agents.

## 2. Results

### 2.1. uPAR Modulators and Their Cancerous Signaling Networks

**Figure 1:**
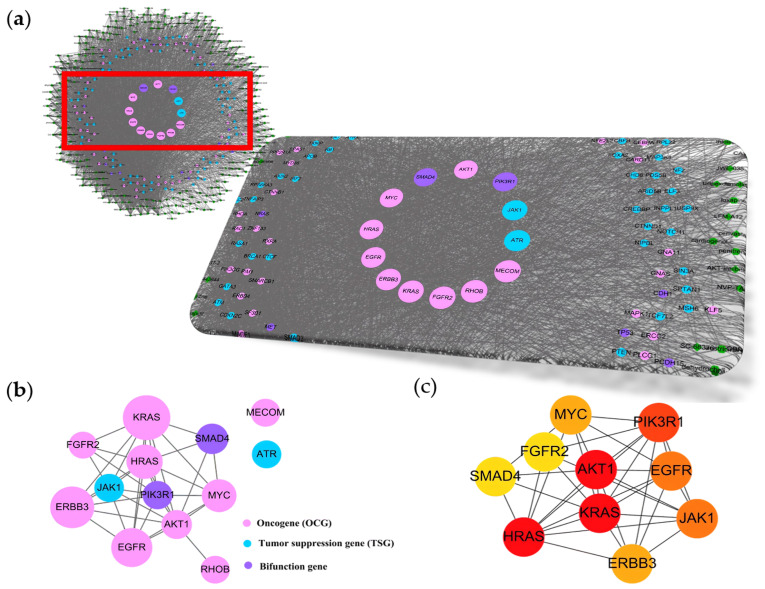
Constructed uPAR modulators–targets networks and module analysis of their interactions. (Transcriptional signaling) (**a**) Compounds–targets network and its amplified view. Dark green nodes represent compounds; other color nodes represent interacted targets being cancer driver genes. Each edge represents the interaction between them. Node size was proportional to its interaction degree. (**b**) The core interactions of this network, which comprised 13 cancer driver genes, was constructed using STRING with PPI score > 0.7. (**c**) The interaction network of top ten uPAR-related cancer driver genes was prepared by Cytohubba (ver. 0.1) in Cytoscape. Node color denotes interaction degree (red for high degree, orange for intermediate degree, and yellow for low degree).

To identify small molecule drug candidates and uPAR-associated targets in human cancer signaling, we proposed that cancer driver genes (CDG) could be hubs to project the crosstalk between uPAR signaling and cancer. Therefore, the first step was to identify uPAR modulators from a comprehensive database, as described in Section 4, to serve as the connectors between uPAR and cancer signaling systems. Based on the data of CMap, which collects the expression profiles of 3848 genes perturbated by 2429 compounds in two ways (i.e., gene knockdown and gene overexpression), 254 uPAR modulators, including 111 stimulators and 143 suppressors, were identified by self-written data extraction scripts. Hypothetically, the interactions between these uPAR modulators and their transcriptional (mRNA) or translational (protein) targets would be comprised by the uPAR signaling networks. Because the natures of transcriptional and translational interactions are different, the construction of the uPAR networks with the protrusion of cancer driver genes were performed as follows:

In transcriptional networking, there were 3848 molecular targets identified, which were co-expressed with the 254 uPAR modulators using the same method of modulator identification (i.e., gene expression score > 0.9), and these targets and modulators were the components of the uPAR transcriptional crosstalk. Then, 299 widely accepted cancer driver genes [10] were introduced, their existence was mapped in the uPAR transcriptional signaling, and 158 of them were found in the network. By relating these associated cancer driver genes and uPAR modulators to one another, a cancerous uPAR compound–target network was built (Figure 1A). The constructed signaling system comprised 411 nodes and 5184 edges, with an average degree (the number of connections or edges the node has to other nodes) of 32.81 nodes per targets and 20.49 edges per compound, respectively. Network analysis results showed that 13 cancer driver targets (i.e., *KRAS*, *ERBB3*, *EGFR*, *HRAS*, *MYC*, *SMAD4*, *AKT1*, *PIK3R1*, *JAK1*, *ATR*, *MECOM*, *RHOB* and *FGFR2*) interacted with more than 90 compounds, forming the core of this cancerous uPAR signaling system, whose average node degree was 5.38. In this core signaling, oncogenes MECOM and RHOB and tumor suppressor gene ATR were linked laterally (Figure 1B). This finding suggested that the 10 cancer driver genes present in the core network might be the essential starters in the modulation of uPAR-mediated functions in cancers (Figure 1C).

**Figure 2:**
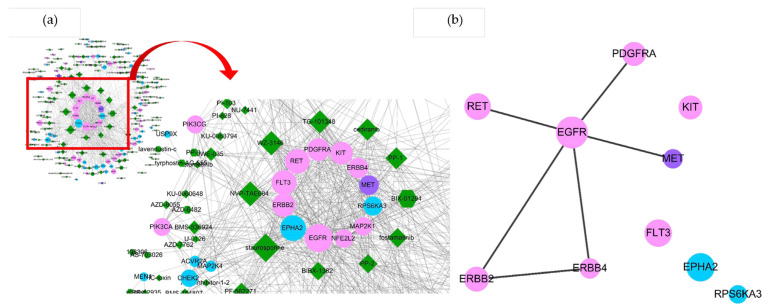
Constructed uPAR modulators–targets network and module analysis of their interactions. (Translational signaling) Node color denotes gene type (pink for oncogene, light blue for tumor suppressor gene, and purple for bifunctional gene) (**a**) Compounds–targets network. Dark green nodes represent compounds; other color nodes represent interacted cancer driver genes. Each edge represents the interaction between them. Node size was proportional to its interaction degree. (**b**) The interaction network of top 10 uPAR-related cancer driver genes were prepared by Cytohubba (ver. 0.1) in Cytoscape.

On the other hand, because the translation from nucleic acid coding to protein coding is known for the existence of expression heterogeneity, the uPAR signaling based on compound–protein and protein–protein interactions could be an alternative route to modulate uPAR-mediated functions. In translational networking, the same 254 uPAR modulators were used to retrieve protein targets from PubChem and similarity ensemble approach (SEA) databases to relay 2186 experimental or putative human protein targets within the uPAR interaction network (Figure 2A). As seen in the core, 12 out of 86 cancer driver targets identified in this networking had the most abundant interactions with uPAR modulators (as ligands). Through analyzing node degree, it was shown that the top 10 cancer driver targets that interacted with uPAR modulators were EGFR, EPHA2, ERBB2, FLT3, RET, PDGFRA, KIT, ERBB4, MET and RPS6KA3 (Figure 2B). Indeed, the EGFR and ERBB families (ERBB2, ERBB3 and ERBB4) were also ranked in the top 10 cancer driver targets in the transcriptional networking, suggesting their roles in uPAR-mediated tumorigenesis processes were highly anticipated.

### 2.2. Essences of uPAR-Mediated Signaling (Table 1) in Cancer Pathways

Cancer is a complicated and heterogeneous diseases. The more the content of the uPAR signaling involved in tumorigeneses is clarified, the higher the chance to develop a novel and successful anti-cancer modality based on modulating this signaling. Therefore, a portion of the uPAR signal network in mediating cancer hallmarks was further extrapolated by counting the involvement of uPAR-related cancer driver genes in common cancer pathways. The network analysis was performed via inputting the cancer driver genes of each uPAR modulator, to map out their existence in the Kyoto Encyclopedia of Genes and Genomes (KEGG) [11] cancer pathways using the service provided by STRING, and the results showed that 10 out of 21 KEGG cancer pathways were comprised of uPAR-related cancer driver genes (203/254, 80%). Among common cancer pathways, these uPAR-mediated pathways, such as MAPK, JAK-STAT, PI3K-Akt, mTOR, focal adhesion, cell cycle, estrogen, VEGF, HIF-1 and apoptosis signaling pathways (Table 1), were known to govern cancer hallmarks, including angiogenesis, cell survival, cell proliferation, invasion and metastasis, suggesting that these cancer pathways not only interacted with uPAR signaling but also embedded druggable targets that could be modulated or affected by uPAR modulators. Moreover, JAK–STAT, PI3K-Akt and MAPK pathways were the main affectable pathways of uPAR modulators, while these three pathways all contained more than 6 of the top 10 uPAR modulator-associated cancer driver genes in their signal transduction routes.

**Table 1:** Network analysis results of the constructed translational uPAR signaling.

Pathway ID	KEGG Pathway Description	Modulators (254)	Counts of Top 10 Driver Genes
		Repetition Rate (Number/254)	
hsa04010	MAPK signaling pathway	**0.97** (246/254)	**7**
hsa04630	JAK-STAT signaling pathway	**0.86** (219/254)	**9**
hsa04151	PI3K-Akt signaling pathway	**0.96** (243/254)	**6**
hsa04150	mTOR signaling pathway	**0.89** (227/254)	**4**
hsa04510	Focal adhesion	**0.82** (208/254)	**4**
hsa04066	HIF-1 signaling pathway	**0.85** (216/254)	**3**
hsa04210	Apoptosis	**0.89** (226/254)	**4**
hsa04370	VEGF signaling pathway	**0.86** (219/254)	**4**
hsa04110	Cell cycle	**0.86** (219/254)	**2**
hsa04915	Estrogen signaling pathway	**0.85** (215/254)	**5**

**Figure 3:**
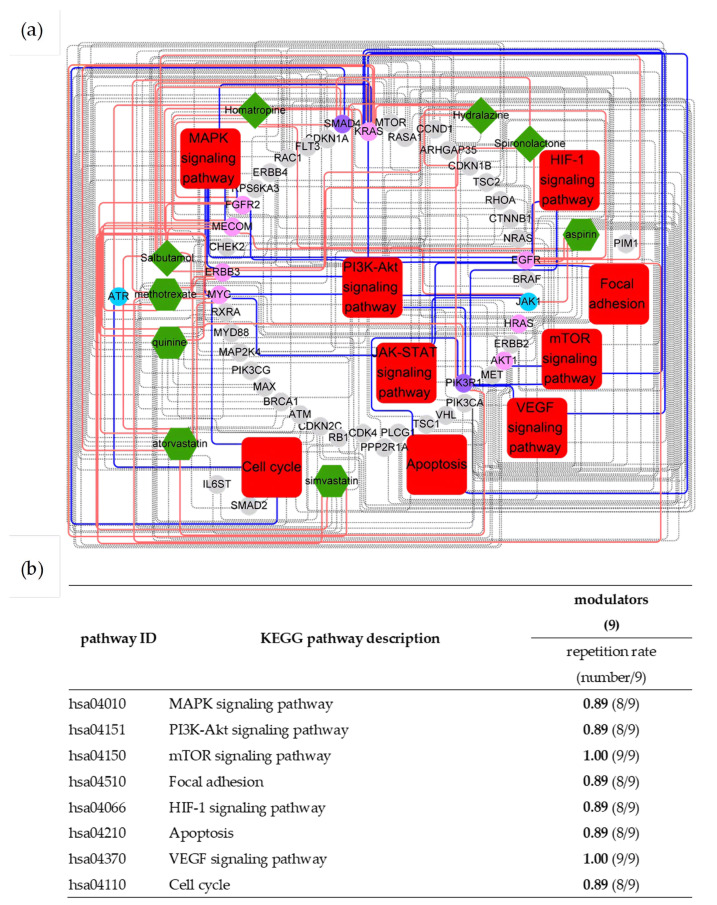
Interactome between real uPAR modulators and their prospective targets. (**a**) Compounds–targets–pathways network. Dark green nodes represent compounds, red nodes mean pathways, and others represent interacted targets being cancer driver genes. (**b**) Main KEGG cancer pathways were affected by real uPAR modulators (from lab testing).

On the other hand, nine real uPAR modulators with proven modulating ability in cell assays [12] were employed to confirm the above analysis results. By using the data of these real modulators to reconstruct the uPAR signaling network for comparison, it was found that the reconstructed network based on nine modulators (Table 2) could interact with 8 out of 10 above-mentioned cancer pathways, including MAPK, PI3K-Akt, mTOR, focal adhesion, cell cycle, VEGF, HIF-1 and apoptosis signaling pathways (Figure 3), suggesting that our approach unbiasedly revealed the tangling essences between uPAR and cancer signaling systems. While some cancer pathways had been previously delineated to interact with the uPAR signaling system, it should be noted that this study was the first study that could address cancerous uPAR targets and pathways so comprehensively.

### 2.3. Virtual Screening of uPAR Modulators by Machine Learning

Based on network analysis results, it was reasoned that uPAR modulators may rep-resent a new class of anti-cancer modalities since they possess the ability to affect can-cerous signaling transduction. Therefore, a virtual screening platform aimed to identify more potential compounds with the ability to modulate the cancerous uPAR signaling for anti-cancer therapy was constructed. By using uPAR expression scores as dependent variables and cancer driver genes as features, a dataset containing 254 uPAR modulators and 185 uPAR-sham compounds with no uPAR activity was prepared from CMap and subjected to a predictive platform model. In construction, seven machine learning algorithms set at tenfold cross-validation were applied to evaluate the prediction performance of the models via indexes, including area under the ROC curve (AUC), accuracy, sensitivity and precision. The results showed that the performance indexes for each algorithm from high to low number of features—i.e., “total features” and “strictly associated features”—were all similar, suggesting the minimum features number for accurate modeling was around 30 and the prediction accuracy could achieve 0.8. (Table 3). Among these models, the neural network model possessed the highest prediction power. To further validate the feasibility of this virtual screening platform, the above-mentioned nine real uPAR modulators were again subjected to classification by these models. It was found that either the SVM model or the neural network model could accurately classify these real modulators with a right pharmacological property (Table 4). Taken together, the above results supported our hypothesis that cancer driver genes were important keystones in uPAR signaling to relay tumorigenesis processes. In summary, our study not only explained the mechanism of action of this cancerous signaling transduction but also provided druggable targets for implication in anti-cancer therapy.

### 2.4. Implication of uPAR Modulators in Anti-Cancer Therapy

In the study, the proposed mechanism of action of uPAR modulators was to affect uPAR-mediated cancer hallmarks, including cell proliferation, migration, angiogenesis and resisting cell death, via modulation of uPAR and its associated downstream cancer driver genes. Compared to conventional uPA/uPAR inhibitors, this type of drug action indeed favors drug discovery in the selection of appropriate drugs, since their effects can be stratified according to the upfront activities on some specific driver genes. For example, uPAR-mediated cancer hallmarks can be further divided into four signaling systems based on composed cancer driver genes, as well as preferred marker genes. (Table 5) As seen, a modulator possessing the activities on uPAR and MYC, an oncogene, could be prone to affecting cell proliferation and survival, whereas one possessing the activities on uPAR and SMAD4, a tumor suppressor gene, could affect angiogenesis and migration.

Furthermore, through surveying in-house and CMap data again, it was verified that stains, including atorvastatin, simvastatin, and lovastatin, ellipticine and pterostilbene, could be potential modulators for anti-cancer purpose, but haloperidol and phenazopyridine could be carcinogenic. (Table 6) Using the pair rule to differentiate modulators’ pharmacological activities in advance, these potential anti-cancer agents could therefore be more precisely applied to treat cancers in situ or in metastasis. This is a good fit with clinical settings where cancer patients are often diagnosed at different progression stages and need regimen options for treating various degrees of malignancy. Thus, the developing uPAR modulators are expected to be a flexible but targeting anti-cancer remedy when used with or without standard chemotherapies.

## 3. Discussion

In this study, the whole uPAR signaling pathway, via linking associated target genes/proteins retrieved from several large public databases (i.e., CMap, PubChem Bio-Assay, SEA, etc.), was successfully constructed. The transformation of this cancerous signaling to a uPAR-centered drug-target network focused on therapeutic application was demonstrated. Through assessing the constructed transcriptional and translational pathways, a predictive machine learning model, using cancer driver genes as its features for identifying so-called uPAR modulators from market drugs, was established. In addition, the mechanism by which the uPAR signaling system changes cancer hallmarks was elucidated. Nevertheless, it is the first study to show there are about 10 cancer pathways associated with uPAR signaling.

The primary function of uPAR is the localization of the proteolytic activity of uPA on the extracellular surface to degrade the extracellular matrix and promote cell migration [19]. Lately, a large body of evidence has implicated that uPAR has an intracellular role in the regulation of various biological processes that contribute to cancer hallmarks. According to comprehensive reviews [4,6], uPAR interacts with more than 42 proteins and behaves as a central mediator in the epithelial–mesenchymal transition (EMT) process relating cell proliferation, differentiation, migration and survival. Crosstalk between uPAR and fMLP receptors (FPRs) regulates cell migration in vitro and in vivo [3,20]. The interaction of CXCR4, which is strongly upregulated in various malignancies, with uPAR-associated proteins drives disseminating cells toward metastatic sites [21], and the complexing of uPA with uPAR regulates EMT to promote pancreatic cancer progression [22]. In addition, the silencing of uPAR depressed hypoxia-induced EMT in multiple cancer cells, but the overexpression of uPAR mimicked EMT under normoxia [23]. Moreover, a positive loop connecting the uPA/uPAR system with TGF-β, where TGF-β could increase the expression of uPA and uPAR, activates plasminogen to plasmin, then triggers latent TGF-β [24]. However, it should be noted that all these studies and reviews could not reveal the whole landscape of uPAR signaling in mediating many physiological and pathological processes. Therefore, in the study, we proposed this pharmaceutical bioinformatics approach to elucidate the detailed crosstalk between uPAR and cancer, to highlight novel anti-cancer targets and/or drugs, as this signaling system is essential in cancer biology [25,26].

In the transcriptional networking, the top 10 cancer driver genes identified to be kin to the uPAR signaling are *AKT1*, *KRAS*, *HRAS*, *PIK3R1*, *JAK1*, *EGFR*, *MYC*, *ERBB3*, *FGFR2* and *SMAD4*. Three of them, *EGFR*, *ERBB3* and *FGFR2*, belong to the class of receptor tyrosine kinases (RTKs), and RTKs are transmembrane proteins that control important cellular processes such as cell growth, survival, and differentiation. It is well documented that dysregulation of RTKs is linked to the pathogenesis of many diseases, notably cancer, making RTKs a therapeutic target for treating tumors [27]. EGFR and ERBB3 both belong to the ERBB family, which were reported by activating the PI3K pathway for transmission signal [28]. The use of monoclonal antibodies (mAbs) or small molecule tyrosine kinase inhibitors (TKIs) to inhibit ERBBs is the most common approach to providing anti-cancer effects. Cetuximab and Panitumumab are FDA-approved mAbs against EGFR, and Erlotinib and Gefitinib are EGFR-TKIs first-line therapies for patients with non-small cell lung cancer (NSCLC). In contrast to their therapeutic efficacy, however, these medicines suffer emerged resistance and rapid tumors regrowth [29]. Previous data indicated that uPAR induced the resistance to anti-cancer agents via the EGFR/p-AKT signaling pathway, as observed in gefitinib-resistant human lung adenocarcinoma cells [30] and vemurafenib-resistant melanoma cells [31].

PIK3R1 and AKT1 both belong to the PI3K pathway, which is the most frequently altered pathway in cancer. PIK3R1 (the code for p85α) was the most abundant isoform to be expressed broadly in normal tissues [32], functioning as a tumor suppressor. The deficiency of PIK3R1-induced hepatocellular carcinogenesis [33] but complete loss of PIK3R1 impaired mouse survival [34]. On the other hand, AKT1, also named threonine kinase 1, was phosphorylated by PI3K as a critical node in the PI3K pathway. AKT1 dysregulation leads to tumorigenesis processes, including cancer cell proliferation, growth and survival [35], tumor angiogenesis [36], recruitment of inflammatory cells required for the tumor microenvironment [37] and resistance to apoptosis [38]. It was previously demonstrated that silencing of uPAR reduced PI3K/AKT downstream signaling activation in rheumatoid arthritis [39] and astrocytoma [40].

KRAS and HRAS are in the RAS/MAPK pathway to relay signals from outside the cell to the nucleus to instruct cell growth, proliferation and differentiation [41]. RAS proteins are believed to be molecular switches and cycle between “on” and “off” conformations by binding to GTP and GDP, respectively [42]. Among the RAS protein family, KRAS is the most frequently mutated protein in human cancer, followed by NRAS and HRAS [42]. It is evident that overexpressed uPAR could be related to oncogenic features such as adhesion in RAS-mutated NSCLC and CRC cells [43].

Except for the above-mentioned seven cancer driver genes, MYC played a role in regulation of protein-coding and non-coding genes to affect cellular functions, which include cell proliferation, differentiation, survival and immune surveillance. In relation to uPAR signaling, MYC downregulated the uPA system to reduce cell motility and invasiveness in gastric cancer [44], while silencing uPAR was associated with decreased c-MYC expression in melanoma and colon cancer [45,46]. On the other hand, JAK1 could behave as an oncogene or tumor suppressor under certain conditions. It played a critical role in different stages of cancer progression [47] and was essential for transduction of IL6-class inflammatory cytokine signaling; it was also involved in mediation of the oncogenic activation of STAT3 in mammary cancer cells [48]. It is documented that the uPAR signaling complex recruited JAK/STAT signaling components to transmit activation signals via STAT1 phosphorylation [49]. Therefore, the potential utility of uPAR signaling in anti-cancer therapy simply cannot exclude the involvement of JAK/STAT signaling. There are some JAK1 inhibitors used in clinics, such as Ruxolitinib, a selective inhibitor of JAK1 and JAK2, for the treatment of myelofibrosis and polycythemia [50]. However, there are limited clinical trials for examining the efficacy of JAK inhibitors on solid tumors so far. Based on the tight tangling between uPAR signaling and cancer, JAK inhibitors might be potential anti-cancer repurposing drugs when used in combination with chemotherapy, radiotherapy, immunotherapy or other targeted agents (e.g., the EGFR inhibitor Erlotinib) [51].

Lastly, SMAD4 as a transcription factor was the central mediator of TGF-β signaling, and it broadly interacted with many classical pathways, such as MAPK, PI3K/AKT and WNT/β-catenin, forming a complex network responsible for a wide range of cellular processes, such as proliferation, differentiation, apoptosis and migration, as well as cancer initiation and development [52]. In cancer signaling, SMAD4 was a tumor suppressor whose deficiency commonly occurred in pancreatic, colorectal, cholangiocarcinoma and many other less common cancers and was associated with pathological stages [53]. Whether SMAD4 plays an essential role in transduction of uPAR signaling remained unsolved, but it was reported that SMAD4 could regulate the expression of PAI-1 [54].

**Figure 4:**
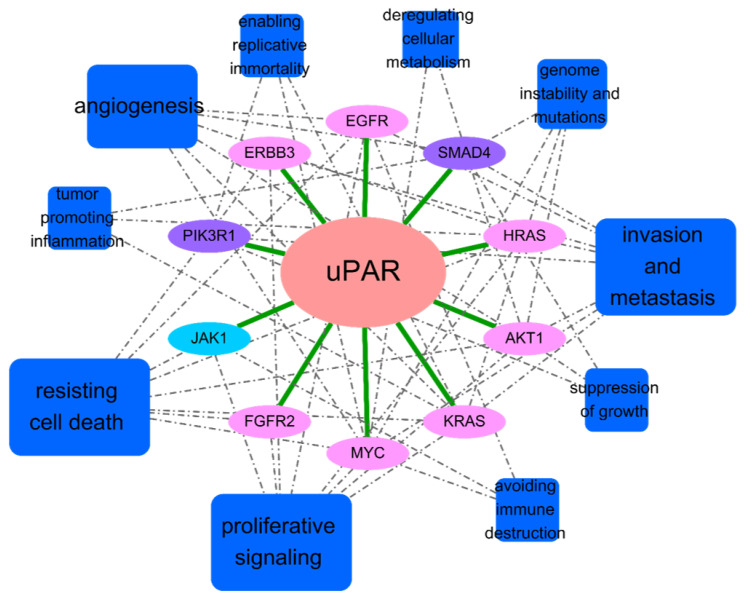
Main cellular functions (cancer hallmarks) and cancer driver genes mediated by the uPAR signaling system. Blue rectangles and colored ovals denote mediated cancer hallmarks and driver genes, respectively.

Indeed, the top ten cancer drug genes could be utilized to derive a uPAR-centered cancer hallmarks map that clearly illustrated the main cellular functions mediated by uPAR signaling (Table 7 and Figure 4). Based on the numbers of connections, it could be identified that angiogenesis, resisting cell death, proliferative signaling, invasion and metastasis would be the target indications when developing uPAR modulators for anti-cancer therapies. This is also in good agreement with empirical experience that indicates an inhibitor blocking the uPA/uPAR signaling pathways may exhibit its function in regulating the EMT process. On the other hand, as indicated in Section 2, the paired expressions of uPAR and some specific cancer driver genes (i.e., MYC and SMAD4) can be utilized to further differentiate drug effects for precise medication to revert cancerous phenotypic change. This use facilitates the drug development process from screening of candidate drugs toward appropriate indications. For example, statins, experimentally proven to be uPAR modulators by us [12], were demonstrated to possess anti-proliferation and anti-metastasis activities at concentrations higher than 10 μM in several studies [12,13,55]. Also, statins were shown to exhibit biphasic angiogenic effects at lower concentrations of 0.1–10 μM [56]. Given MYC and SMAD4 are known to be modulated by statins [57,58], the phenotypic switch caused by high and low doses of the same uPAR modulator may become an interesting research topic to elucidate whether these cancer driver genes could also serve as surrogate markers in anti-cancer therapies.

In the analysis of translational networking, RTKs were again found to play important roles in the transduction of uPAR signaling to mediate cellular functions, suggesting our approach was useful in the elucidation of complicate cancer biology whether it is genomic data or not. On the other hand, RPS6KA3, identified as one of the top 10 cancer driver targets in the uPAR translational networking, belongs to a family called ribosomal S6 kinases (RSKs). It worked as a downstream substrate of extracellular signal-regulated kinases (ERK) to function in the RAS/MAPK pathway, regulating many physical and pathological processes [59,60]. In the presence of RPS6KA3 boosters [61], cell migration was significantly decreased while silencing uPAR. Nevertheless, this is the first time that the uPAR signaling system, via bridging two intracellular kinase systems, i.e., RTKs and RSKs, was shown to execute and exhibit its functions.

Moreover, the pathway enrichment analysis results suggested that approximately 80% of uPAR-mediated signaling crosstalked to the identified 10 cancer pathways, where the MAPK and PI3K-AKT pathways were evident to be the most augmented. The involvement of these two pathways in cancer was likely through the complexing of uPAR with integrins/RTKs, as discussed above. The MAPK pathway was tightly regulated by phosphatases to communicate bidirectionally with other pathways, including the PI3K/AKT/m-TOR pathway. Dysregulation of the MAPK and PI3K-AKT pathways could bring uncontrolled cell proliferation, and such a machinery was already labeled as a part of the cancer hallmarks [62]. Given the close relationships of uPAR with these identified cancer pathways, our study should help illustrate which druggable targets in uPAR signaling are useful for reversing cancer hallmarks. Consequently, the potential uPAR modulator proposed by this study could be a new class of anti-cancer agents for effectively treating cancers, since how these compounds change cancer hallmarks, such as via sustaining proliferative signaling, resisting cell death, inducing/accessing vasculature and activating invasion and metastasis, etc., is much clearer now.

Finally, a machine learning screening platform based on cancer driver genes for the identification of potential uPAR modulators was established. This model could be used for in silico drug screening, but also for mechanism of action elucidation based on the intracellular uPAR signaling system, which was composed of about 30 cancer driver genes that impelled its versatile functionalities. While market drugs could have more certain safety profiles than investigating compounds, our approach could offer the profound benefits as finding a candidate and deciphering its mechanism of action at the same time. Compared to conventional uPA/uPAR inhibitor development, this indeed provides a paved way to efficiently identify small molecule anti-cancer agents that are either new synthetic compounds or old market drugs.

## 4. Materials and Methods

**Figure 5:**
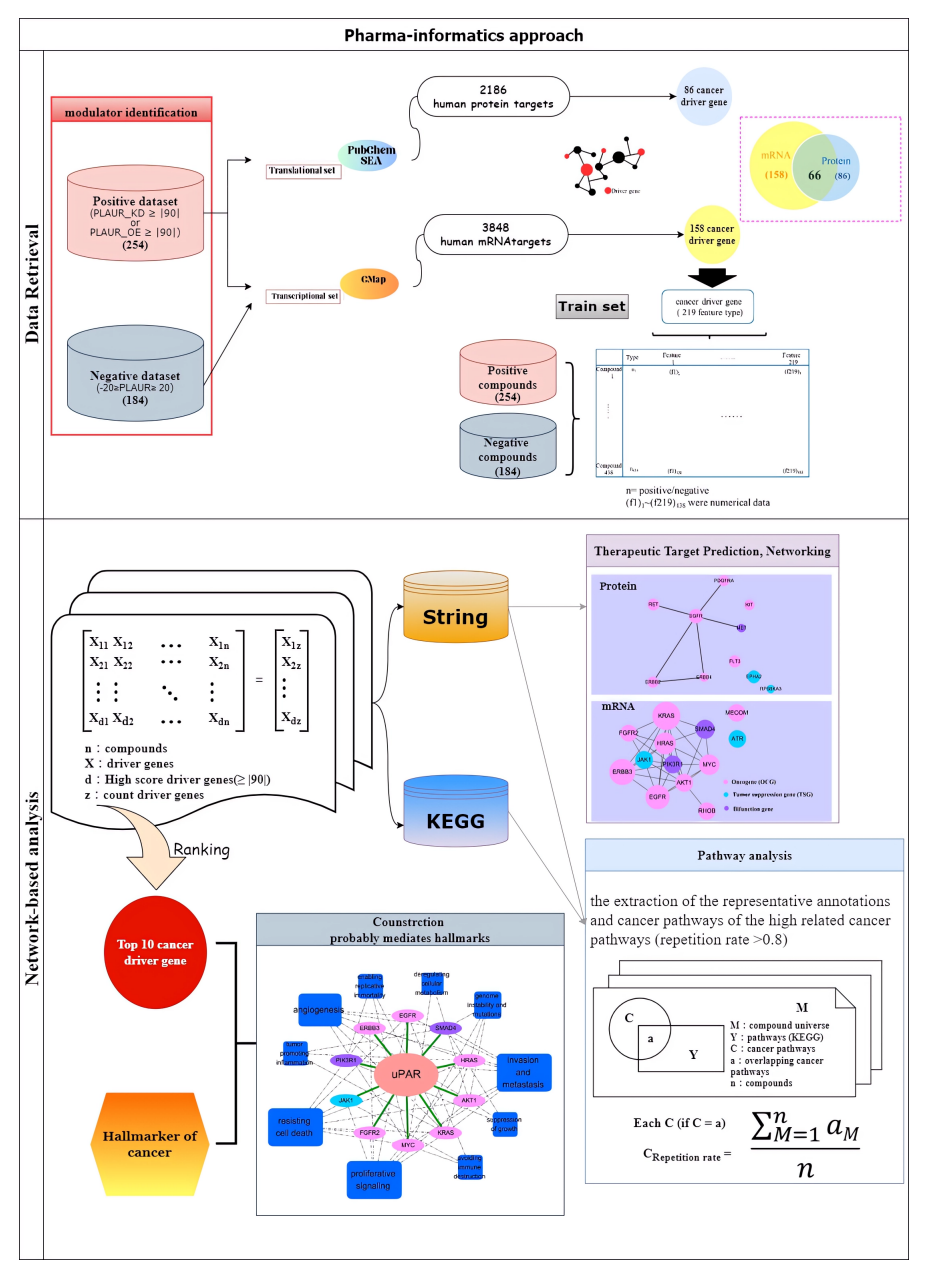
Workflow of this integrated pharmaceutical bioinformatics study.

In the study, an integrated pharmaceutical bioinformatics approach (Figure 5) that combined modulator identification, driver gene ontology networking, protein targets prediction and networking, pathway analysis and uPAR modulator screening platform construction was employed to address the crosstalk between uPAR signaling and cancer, as well as to explore the druggable targets in uPAR signaling for potential anti-cancer applications. Interactome data, including transcriptional (mRNA), translational (protein) and perturbation (modulator) data retrieved from multiple public databases (Table 8), were used to mine the uPAR networking and to decipher its essences in modulating cancer. Cancer driver genes used for this framework study were adopted mainly from authoritative literature sources [10]. Since the first layer data (uPAR modulator) were generated from a database covering merely 3848 targets, the mapping ratio of driver to non-driver genes was around 1:12.

### 4.1. Modulator Identification

To address the crosstalk between uPAR signaling and cancer, we first used the gene perturbation data from the Connectivity Map (CMap) [65] database to identify compounds, namely the uPAR modulator, as its perturbation score of uPAR gene exceeding 0.9. In total, 2429 compounds collected in CMap were subjected to modulator extraction using self-written computer scripts.

### 4.2. Target/Cancer Driver Gene Ontology Networking

Two procedures were used to map out the uPAR signaling with cancer driver genes, the identification of uPAR co-activated cancer driver genes from the CMap perturbation data and connection of all these identified targets, to assemble the uPAR–driver genes network. The identification of active (co-expressed) cancer driver genes from the perturbation data of uPAR modulators was the same as that used above, in the identification of uPAR modulators (i.e., perturbation score exceeding 0.9), whereas the network construction and analysis of uPAR signaling was performed by means of Cytoscape 3.8.2, which is a popular visualization interface for probing complicated biological interactions [66]. The type of networking is called the uPAR transcriptional network.

### 4.3. Therapeutic Target Prediction, Networking and Pathway Analysis

To interpret the possible functional interaction between uPAR signaling and cancer, an alternative uPAR-mediated target–pathway (T-P) network was also constructed in this study. This involved retrieving the protein targets that experimentally or putatively interacted with the uPAR modulators, from the PubChem BioAssay [63] and similarity ensemble approach (SEA) [64] databases, and filtering out non-cancer driver protein targets; the enriched T–P network of the translational uPAR-signaling was constructed by means of STRING-DB (11.0), with targets tagged Homo sapiens taxonomy and confidence scores greater than 0.7. For pathway analysis, the extraction of the representative annotations and cancer pathways of the top 10 clusters was performed by using CytoHubba [67]. This is called the uPAR translational network.

### 4.4. Data Mining and Predictive Model Construction

To further explore the usefulness of uPAR modulator in cancer therapy, an in silico screening platform based on the expressions of cancer driver genes as features and modulator activity as dependent variables was constructed. Except for the 254 uPAR modulators identified in the above process, the negative dataset, including 185 uPAR-sham modulators, was prepared from the CMap compounds pool with uPAR perturbation scores between −0.2 to 0.2. The model construction was performed by machine learning using seven algorithms implemented in Orange [68], which included logistic regression, support vector machine (SVM), random forest, naïve Bayes, neural network, gradient boosting and k-nearest neighbors (kNN). In this procedure, 70% of the compounds were set for training and 30% for testing using a relevance-based feature selection (CFS) algorithm and tenfold cross-validation for model construction. A confusion matrix was applied to calculate the sensitivity, precision and overall accuracy of each algorithm.

## 5. Conclusions

In the study, the cancer driver genes and/or targets that compose the uPAR signaling system were successfully identified. The crosstalk between uPAR signaling and cancer pathways could be illustrated through kinase signaling (i.e., RTKs and RSKs) and intracellular cancer signal pathways, such as MAPK and PI3K-AKT pathways, to communicate and to affect cellular phenotypes (i.e., cancer hallmarks) during tumorigenesis and progression. With elucidation of the complicate networking between uPAR modulation and cancer biology, our results nevertheless provide the strongest context for further developing uPAR modulators as a new class of anti-cancer medicines.

## Figures and Tables

**Table 2:** Real uPAR modulators and their affectable cancer driver genes.

Active Modulators	Cancer Driver Genes
**Suppression**	
Homatropine	**AKT1**, ARHGAP35, ATR, AXIN2, BTG2, CCND1, CDKN1B, CHEK2, DICER1, DNMT3A, **EGFR**, EPAS1, ERBB2, **ERBB3**, FAT1, FBXW7, **FGFR2**, **HRAS**, **JAK1**, **KRAS**, MECOM, MSH6, MYD88, PDS5B, PMS1, RAC1, RASA1, RET, RHOA, RHOB, RXRA, SF3B1, **SMAD4**, TCF7L2, ZBTB20, ZFP36L1
Hydralazine	ARHGAP35, B2M, BTG2, CCND1, CDK4, CDKN2C, CHEK2, **EGFR**, **ERBB3**, FLT3, **KRAS**, MET, NIPBL, PIK3CA, **PIK3R1**, PLCB4, PLCG1, RAC1, RASA1, RET, RPS6KA3, **SMAD4**, SMARCB1, WT1, ZBTB20
Salbutamol	ARHGAP35, ATR, BRAF, CCND1, CTNNB1, **EGFR**, **ERBB3**, ERBB4, **FGFR2**, **HRAS**, IDH2, **KRAS**, MAP2K4, MECOM, MEN1, MTOR, **MYC**, PRKAR1A, RAC1, RHOA, SF3B1, **SMAD4**, SMARCB1, TRAF3
Spironolactone	ARHGAP35, AXIN2, CDKN2C, **EGFR**, ERBB2, **ERBB3**, FOXQ1, GNA13, **HRAS**, NRAS, RHOB, **SMAD4**, TSC2, ZFP36L2
**Stimulation**	
Aspirin	APOB, ARHGAP35, ATXN3, AXIN2, B2M, CHEK2, **EGFR**, ERBB4, FLT3, FOXQ1, GNA13, GNAS, **HRAS**, IDH1, **JAK1**, MAP2K4, MECOM, MET, **MYC**, PIK3CA, **PIK3R1**, POLRMT, PPP2R1A, PSIP1, RASA1, RB1, RET, RHOB, RPL22, SMARCB1, TCF7L2, TSC1, TSC2, WT1
Atorvastatin	ARID5B, ATM, ATR, BRCA1, CDKN1A, CDKN2C, CTNND1, **ERBB3**, FLT3, IL6ST, IRF2, **KRAS**, MECOM, MEN1, MTOR, **MYC**, NRAS, **PIK3R1**, **PIK3R1**, PIM1, RHOB, RPS6KA3, RXRA, SF1, SMAD2, TSC2, WT1, ZBTB20, ZFP36L1, ZMYM2, ZNF133
Methotrexate	**AKT1**, ARHGAP35, ATR, AXIN2, BRCA1, CHEK2, **EGFR**, **ERBB3**, **KRAS**, MAX, MECOM, MEN1, MTOR, **MYC**, PIK3CA, PIK3CG, RHOA, RXRA, TCF12, TCF7L2
Quinine	ATM, BRCA1, CDKN2C, CHEK2, **ERBB3**, ERBB4, **FGFR2**, GNAS, **HRAS**, IRF6, MAP2K4, MAX, MECOM, MET, MTOR, NRAS, PIK3CG, **PIK3R1**, PRKAR1A, RAC1, RB1, SMARCB1, TNFAIP3, TSC2, TXNIP, USP9X, VHL, ZFP36L2
Simvastatin	CDK4, CDKN1A, CDKN2C, **ERBB3**, IL6ST, **KRAS**, MAP2K4, **MYC**, MYD88, RAC1, RHOB, RXRA, TXNIP, ZFP36L1, ZMYM2

Top 10 cancer driver genes identified in this study are highlighted in bold.

**Table 3:** Performance of various machine learning algorithms on model construction.

Classifier	Performance			
	AUC	Accuracy	Sensitivity	Precision
*Total Features* (**218**)				
kNN	0.850	0.727	0.727	0.717
SVM	**0.921**	0.775	0.775	0.770
Random Forest	0.853	0.702	0.702	0.692
**Neural Network**	0.920	**0.788**	**0.788**	**0.784**
Naive Bayes	0.868	0.698	0.698	0.708
Logistic Regression	0.912	0.778	0.778	0.776
Gradient Boosting	0.891	0.730	0.730	0.722
*Strictly Associated Features* (**31**)				
kNN	0.872	0.738	0.738	0.731
SVM	**0.917**	0.774	0.774	0.770
Random Forest	0.877	0.728	0.728	0.720
**Neural Network**	0.913	**0.778**	**0.778**	**0.779**
Naive Bayes	0.905	0.747	0.747	0.748
Logistic Regression	0.896	0.749	0.749	0.749
Gradient Boosting	0.905	0.755	0.755	0.750

The number of features for prediction models. At each feature selected set, the highest prediction performances are highlighted in bold.

**Table 4:** Classification results of real modulators using virtual screening platform.

		*Total Features* (218)		*Strictly Associated Features* (31)	
		SVM	Neural Network	SVM	Neural Network
uPAR activity		9	9	9	9
No activity		0	0	0	0

**Table 5:** Stratification of uPAR-mediated cancer hallmarks, driver genes and preferred markers.

Hallmarks	Driver Gene	Preferred Marker
**proliferative signaling**	AKT1, EGFR, ERBB3, FGFR2, HRAS, JAK1, KRAS, MYC	MYC
**resisting cell death**	AKT1, EGFR, ERBB3, FGFR2, HRAS, JAK1, KRAS, MYC	MYC
**angiogenesis**	AKT1, EGFR, HRAS, KRAS, SMAD4	SMAD4
**invasion and metastasis**	AKT1, EGFR, ERBB3, HRAS, KRAS, MYC, PIK3R1, SMAD4	PIK3R1, SMAD4

**Table 6:** Identified uPAR modulators and their possible implications.

Predicted Drug	CMap Expression Score *			Drug Effects	Referred Validation
	uPAR	MYC	SMAD4		
Statins (atorvastatin)	88.31(KD)	99.15(KD)		Anti-cancer	In-house data, [12,13]
Ellipticine	91.97(KD)	90.64(KD)		Anti-cancer	[14]
Pterostilbene	95.19(KD)	94.29(KD)		Anti-cancer	[15,16]
**haloperidol**	94.07(OE)		98.59(KD)	Carcinogenic	[17]
**phenazopyridine**	92.97(OE)		94.41(KD)	Carcinogenic	[18]

* Data retrieved from CMap; KD denotes knock down and OE denotes over express.

**Table 7:** Associations of top 10 uPAR-related cancer driver genes to cancer hallmarks.

Driver Gene	Gene Description	Hallmarks
AKT1	AKT serine/threonine kinase 1	proliferative signaling, evading growth suppressors, invasion and metastasis, angiogenesis, resisting cell death, deregulating cellular metabolism, genome instability and mutations
EGFR	ErbB (epidermal growth factor) receptor family, epidermal growth factor receptor	proliferative signaling, avoiding immune destruction, invasion and metastasis, angiogenesis, resisting cell death, deregulating cellular metabolism
ERBB3	ErbB (epidermal growth factor) receptor family, v-erb-b2 erythroblastic leukemia viral oncogene homolog 3	proliferative signaling, invasion and metastasis, resisting cell death
FGFR2	Type V RTKs: FGF (fibroblast growth factor) receptor family, fibroblast growth factor receptor 2	proliferative signaling, resisting cell death
HRAS	RAS subfamily, v-Ha-ras Harvey rat sarcoma viral oncogene homolog	proliferative signaling, tumor-promoting inflammation, invasion and metastasis, angiogenesis, genome instability and mutations, resisting cell death, avoiding immune destruction
JAK1	Janus kinase (JakA) family, Janus kinase 1	proliferative signaling, avoiding immune destruction, resisting cell death
KRAS	RAS subfamily, v-Ki-ras2 Kirsten rat sarcoma viral oncogene homolog	proliferative signaling, enabling replicative immortality, tumor-promoting inflammation, invasion and metastasis, angiogenesis, resisting cell death, deregulating cellular metabolism
MYC	Basic helix-loop-helix proteins, v-myc myelocytomatosis viral oncogene homolog	proliferative signaling, angiogenesis, avoiding immune destruction, genome instability and mutations, deregulating cellular metabolism, resisting cell death, invasion and metastasis, enabling replicative immortality
PIK3R1	Phosphatidylinositol kinases, phosphoinositide-3-kinase, regulatory subunit 1 (alpha)	evading growth suppressors, enabling replicative immortality, invasion and metastasis
SMAD4	SMADs, SMAD family member 4	evading growth suppressors, tumor-promoting inflammation, invasion and metastasis, angiogenesis, genome instability and mutations

**Table 8:** Publicly accessible databases used in this pharmaceutical bioinformatics study.

Resource	Description	Website	Ref.
Translational (protein)			
PubChem	A web-based informatics environment for data from small molecules and their biological activities.	https://pubchem.ncbi.nlm.nih.gov/ (accessed on 1 July 2023)	[63]
Similarity ensemble approach (SEA)	An open resource related to proteins based on the set-wise chemical similarity among their ligands.	http://sea.bkslab.org/ (accessed on 1 July 2023)	[64]
**Transcriptional (gene)**			
Connectivity Map (CMap)	A public catalog of gene expression data collected from human cells treated with chemical compounds and genetic reagents	https://clue.io/cmap (accessed on 1 July 2023)	[65]
KEGG	A curated database collecting comprehensive data including genes, reactions, pathways, drugs and diseases, for studying functions and utilities of the biological systems	http://www.kegg.jp/kegg/ (accessed on 1 July 2023)	[11]

## Data Availability

All data were retrieved from publicly accessible databases, as indicated in Table 8.
